# Dimensional psychiatry: reward dysfunction and depressive mood across psychiatric disorders

**DOI:** 10.1007/s00213-014-3662-7

**Published:** 2014-06-29

**Authors:** Claudia Hägele, Florian Schlagenhauf, Michael Rapp, Philipp Sterzer, Anne Beck, Felix Bermpohl, Meline Stoy, Andreas Ströhle, Hans-Ulrich Wittchen, Raymond J. Dolan, Andreas Heinz

**Affiliations:** 1 Department of Psychiatry and Psychotherapy, Campus Charité Mitte, Charité – Universitätsmedizin Berlin, Charitéplatz 1, 10117 Berlin, Germany; 2 Max Planck Institute for Human Cognitive and Brain Sciences, Leipzig, Germany; 3 Social and Preventive Medicine, University of Potsdam, Potsdam, Germany; 4 Berlin School of Mind and Brain, Berlin, Germany; 5 Institute of Clinical Psychology and Psychotherapy, Technische Universität Dresden, Dresden, Germany; 6 Wellcome Trust Centre for Neuroimaging, Institute of Neurology, University College London, London, WC1N 3BG UK; 7 Visiting Einstein Fellow, Mind and Brain Centre, Humboldt University, Berlin, Germany

**Keywords:** Dimensional, fMRI, Reward system, Ventral striatum, Monetary incentive delay task, Depressive symptoms

## Abstract

**Rationale:**

A dimensional approach in psychiatry aims to identify core mechanisms of mental disorders across nosological boundaries.

**Objectives:**

We compared anticipation of reward between major psychiatric disorders, and investigated whether reward anticipation is impaired in several mental disorders and whether there is a common psychopathological correlate (negative mood) of such an impairment.

**Methods:**

We used functional magnetic resonance imaging (fMRI) and a monetary incentive delay (MID) task to study the functional correlates of reward anticipation across major psychiatric disorders in 184 subjects, with the diagnoses of alcohol dependence (*n* = 26), schizophrenia (*n* = 44), major depressive disorder (MDD, *n* = 24), bipolar disorder (acute manic episode, *n* = 13), attention deficit/hyperactivity disorder (ADHD, *n* = 23), and healthy controls (*n* = 54). Subjects’ individual Beck Depression Inventory-and State-Trait Anxiety Inventory-scores were correlated with clusters showing significant activation during reward anticipation.

**Results:**

During reward anticipation, we observed significant group differences in ventral striatal (VS) activation: patients with schizophrenia, alcohol dependence, and major depression showed significantly less ventral striatal activation compared to healthy controls. Depressive symptoms correlated with dysfunction in reward anticipation regardless of diagnostic entity. There was no significant correlation between anxiety symptoms and VS functional activation.

**Conclusion:**

Our findings demonstrate a neurobiological dysfunction related to reward prediction that transcended disorder categories and was related to measures of depressed mood. The findings underline the potential of a dimensional approach in psychiatry and strengthen the hypothesis that neurobiological research in psychiatric disorders can be targeted at core mechanisms that are likely to be implicated in a range of clinical entities.

**Electronic supplementary material:**

The online version of this article (doi:10.1007/s00213-014-3662-7) contains supplementary material, which is available to authorized users.

## Introduction

To date, neurobiological research has identified a variety of dysfunctions implicated in psychiatric disorders, e.g., anhedonia or disturbances in cognition and memory; however, these do not necessarily provide a neurobiological validation of existing disorder categories (Heinz 2002; Robbins et al. 2012; van Os and Kapur 2009). Clinical categories such as “schizophrenia” or “major depressive disorder” (MDD) are complex phenotypes composed of patterns of symptoms with diverse neurobiological signatures (van Os and Kapur 2009). It has been suggested for some time that psychiatric disorders should be separated into their components for diagnostic and treatment purposes, as it is often done in somatic disorders (Heinz 2002; Van Praag et al. 1990). Such a dimensional approach tries to identify neurobiological correlates of core psychological mechanisms, which are often observed in different combinations and various severities in psychiatric disorders (Insel et al. 2010). We and others have suggested that a dimensional approach can be applied across nosological boundaries in major psychiatric disorders and used to identify neurobiological signatures of core (dys)functions, such as positive and negative moods as well as learning from reward and punishment (Insel et al. 2010; Watson and Clark 1988). This approach can be promising for the understanding of psychiatric symptoms and for the development of new treatment strategies (Canli and Lesch 2007; Insel 2012; Robbins et al. 2012). For example, several studies focused on serotonergic (dys)function in healthy participants and across psychiatric disorders, as well as on neurobiological correlates of (an)hedonia in schizophrenia, depression, and alcohol dependence (Canli et al. 2002; Hariri et al. 2002; Heinz et al. 1994, 2005a). With respect to dopaminergic neurotransmission, it was originally hypothesized that dopamine release is associated with the rewarding (pleasurable and hence hedonic) effects of the drug or action that caused dopamine stimulation (Wise 1988). However, based on primate and rodent studies, it was suggested that phasic dopamine release mediates the motivational state of wanting rather than the hedonic pleasure of enjoying a reward (Robinson and Berridge 1993). We and others observed that dopamine release was associated both with positive mood and with craving for reward, while dopamine dysfunction was associated with motivational impairment (apathy) more strongly than with the inability to enjoy pleasure (anhedonia) (Drevets et al. 2001; Heinz et al. 1998; Schmidt et al. 2001).

Animal experiments point to the ventral striatum (VS) as a key area coding reward anticipation and feedback. Functional paradigms to study reward anticipation and learning from reinforcement have been developed on the basis of a computational account of dopamine discharge patterns elicited by violations of expectations, e.g., by unexpected cues predicting reward and by prediction errors due to unexpected reward feedback (Knutson et al. 2001b; Montague et al. 2004; Schultz 1998). Studies in nonhuman primates have indicated that unexpected reward or the appearance of a reward-predicting cue lead to phasic activation of dopaminergic projections including those to the ventral striatum (Schultz et al. 1997). These findings have been translated into an fMRI paradigm, the so-called “Monetary Incentive Delay (MID) task” by Knutson et al. (2000). An increase in firing of dopaminergic neurons is thought to change activation of the ventral striatum and thus affect the blood oxygen level-dependent (BOLD) signal measured via fMRI. In fact, the MID task is known to robustly activate the VS during reward anticipation and to a lesser degree also during anticipation of loss (Knutson et al. 2001a, b), and this has been shown to significantly correlate with key dopamine indicators such as release and synthesis capacity (Schlagenhauf et al. 2013; Schott et al. 2008).

Applying the MID task to a variety of psychiatric disorders was inspired by the idea that dopaminergic dysfunction can affect motivation and mood in several disorders such as major depression, schizophrenia, and alcohol dependence (Bjork et al. 2012; Hasler et al. 2009; Knutson et al. 2008; Nielsen et al. 2012; Simon et al. 2010) (see supplement Table 1). Our own group observed blunted ventral striatal activation elicited by reward-predicting cues in these diagnostic groups compared to healthy controls, and mixed results or no significant group differences in patients with mania or attention deficit/hyperactivity disorder compared to healthy controls (Beck et al. 2009; Bermpohl et al. 2010; Juckel et al. 2006a, b; Schlagenhauf et al. 2008, 2009; Stoy et al. 2012; Strohle et al. 2008; Wrase et al. 2007). These observations suggest that blunted ventral striatal activation during reward anticipation may figure prominently in disorders associated with negative mood states such as schizophrenia, alcohol dependence, and depression, but may not be present in disorders characterized by elevated mood such as mania. The recently revived interest in the dimensional approach (Insel et al. 2010) as well as a study of reward anticipation in adolescents (Bebko et al. 2014) encouraged us to compare dysfunction of the reward system across different psychiatric disorders by pooling our data and enlarging our sample with further subjects. Data (*n* = 184) were available in five different psychiatric disorders (alcohol dependence, schizophrenia, major depressive disorder, acute manic episode of bipolar disorder, and attention deficit/hyperactivity disorder (ADHD)), as well as healthy controls. All subjects underwent scanning with the same MRI scanner using the same protocol and the same scores to assess negative mood (i.e., the Beck Depression Inventory (BDI (Beck et al. 1997)) and the State-Trait Anxiety Inventory (STAI (Spielberger et al. 1970)). According to the literature mentioned above, we hypothesized to observe reduced ventral striatal activation in patients with schizophrenia, alcohol dependence, and major depression (but not with acute manic episode or ADHD) during reward anticipation. We also explored whether negative mood states such as anxiety or depression correlated with impaired VS activation during reward anticipation across diagnostic groups.

## Methods

### Subjects

One hundred thirty-three patients and 54 healthy volunteers participated in this study. Patients were recruited from the inpatient and outpatient center of the Department of Psychiatry and Psychotherapy, Charité-Universitätsmedizin Berlin, Campus Charité Mitte. Healthy controls were recruited from the local community by advertisement. Ethical approval was provided by the local ethics committee, and written informed consent was obtained from all participants after complete description of the study.

All participants were right-handed, as assessed with the Edinburgh Handedness Inventory (Oldfield 1971). Exclusion criteria were current neurological or severe medical disorders, history of head injury resulting in loss of consciousness, and age below 18 or above 65 years. Healthy volunteers had no personal or family history of any psychiatric axis I or II disorder as assessed with the Structured Clinical Interview for DSM-IV disorders (SCID I/II; (First et al. 1997, 2001)) and were not under psychotropic medication.

Patients were diagnosed according to ICD-10 and DSM-IV (assessed with the SCID I and II). Only patients with a single axis I disorder from the following five disorder groups were included: alcohol dependence (ICD-10: F10.2), schizophrenia (ICD-10: F20.0), major depressive disorder (ICD-10: F32.1, F32.2, F33.1, F33.2), bipolar disorder with acute manic episode (ICD-10: F31.1), and attention deficit/hyperactivity disorder (ADHD; ICD-10: F90.0), respectively. All patients were without a personal history of comorbid psychiatric disorders (SCID I/II), except for nicotine dependence. Disorder-specific comparisons between subgroups of patients and healthy controls (altogether *n* = 95) have previously been published (Beck et al. 2009; Bermpohl et al. 2010; Juckel et al. 2006a, 2012; Schlagenhauf et al. 2008, 2009; Schmack et al. 2008; Stoy et al. 2011, 2012; Strohle et al. 2008; Wrase et al. 2007). Here, we report an increased sample of 133 patients and 54 controls. For a detailed description of the sample as well as specific, disease-related scores, please see Tables 1 and 2. Three subjects (one patient with alcohol dependence, two with schizophrenia) were identified as outliers with regard to the mean effect size in the VS region of interest (ROI) for the main contrast “anticipation of gain *minus* neutral cues” with a *z* value of >3 and therefore excluded. The final group consisted of 54 healthy volunteers (13 women, mean±STD 37.7 ± 11.1 years) and 130 patients (32 women, 36.7 ± 10.4 years) (see Table 1).

**Table 1:** Group description

	Healthy controls	Alcohol dependence	Schizophrenia	MDD	Bipolar disorder manic episode	ADHD	Study population	Between-group differences^a^	
								Test	Significance
No. of subjects	54	26	44	24	13	23	184		
Males/females	41/13	25/1	27/17	17/7	8/5	21/2	139/45	χ2 = 15.4	0.009
Mean age in years	37.7 ± 11.1	43.3 ± 7	34.2 ± 9.8	40.1 ± 11.6	36 ± 13	30.7 ± 6.2	37 ± 10.6	*F*_5_ = 5.1	<0.001
Mean duration of illness in years	Not applicable	13 ± 8.6	4.8 ± 5.6	7.8 ± 8.4	12.3 ± 10.4	22.3 ± 6.3		*F*_4_ = 18.4	<0.001
Smoker (male/female)	26 (18/8)	25 (24/1)	29 (19/10)	12 (8/4)	8 (6/2)	14 (12/2)	114 (87/27)	χ^2^ = 18.9	0.002
Medication	None	Free of psychotropic medication at time of scanning	16 none, 28 antipsychotics (mostly 1st generation antipsychotics; mean CPE mg 405 ± 297)	Free of psychotropic medication at time of scanning	1 none, 12 diverse (1–3 substances; including mood stabilizers, benzodiazepines and antipsychotics (mean CPE mg 512 ± 337)	None			
No. of BDI	49	18	29	21	Not applicable	15	132	χ^2^ = 46.9	<0.001
BDI mean score	3.4 ± 3.6	10.7 ± 6.9	14.7 ± 10.8	24.3 ± 9.7	Not applicable	7.8 ± 6.3	10.7 ± 10.4	*F*_4_ = 31.3	<0.001
No. of STAI	43	19	Not applicable	16	11	15	104	χ^2^ = 75.2	<0.001
STAI mean score	33 ± 8.3	41.4 ± 11.9	Not applicable	45.6 ± 9.7	40.6 ± 9.6	40.2 ± 10.2	38.3 ± 10.6	*F*_4_ = 6.3	<0.001
Mean RT in ms	282 ± 87	294 ± 111	377 ± 166	247 ± 57	310 ± 137	274 ± 105	303 ± 123	*F*_5_ = 5.4	<0.001

*MDD* major depressive disorder, *ADHD* attention deficit/hyperactivity disorder, *CPE* chlorpromazine equivalents, *BDI* Beck Depression Inventory, *STAI* State-Trait Anxiety Inventory, *RT* reaction time across all cue conditions during the MID task

^a^For nonparametric test, we used the Kruskal-Wallis test (*df* = 5); for parametric tests, an analysis of variance was computed

**Table 2:** Specific diagnosis-related scores

Diagnosis	Psychopathological measurement scale	Mean±standard deviation
Alcohol dependence	Alcohol Dependence Scale (*Severity of Alcohol Dependence* (Skinner and Horn 1984))	20.7 ± 6.7
	Obsessive Compulsive Drinking Scale (*Severity of Alcohol Craving* (Anton 2000))	20.9 ± 6
Schizophrenia	Positive and Negative Syndrome Scale (PANSS (Kay et al. 1987))	85.7 ± 27.2
Major depressive disorder (MDD)	Hamilton rating Scale for Depression (HRSD-21 (Hamilton 1960))	20.4 ± 4.1
Bipolar disorder, acute manic episode	Young Mania Rating Scale (YMRS (Young et al. 1978))	19.6 ± 6.4
Attention deficit/hyperactivity disorder (ADHD)	Conner’s Adult ADHD Rating Scale (Conners et al. 1999)	64.1 ± 14.4

Ratings of subjective depressive symptoms with the BDI (Beck et al. 1997) and ratings of subjective anxiety symptoms with the STAI (Spielberger et al. 1970) were assessed across diagnostic groups. Altogether, in 83 patients (no patients with acute manic episode included) and 49 controls, BDI ratings were available, and STAI ratings were available in 61 patients (no patients with schizophrenia included) and 43 controls. We compared participants whose BDI was available with participants whose BDI had not been assessed to rule out any differences between them, using a MANOVA with “existing BDI” coded as “0” and “missing BDI” coded as “1.” Likewise, we controlled for differences between participants with and without STAI.

### Monetary incentive delay task

**Fig. 1:**
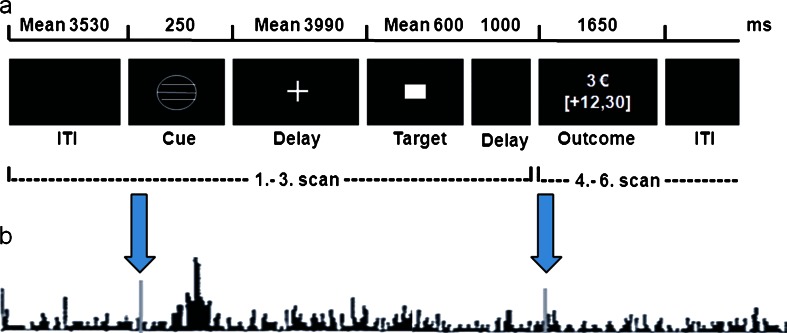
Experimental paradigm “monetary incentive delay task”. (*a*) Trial structure for a representative successful gain (3 €) trial. (*b*) Theoretical background: phasic dopamine outburst at the time of a reward-predicting cue and no phasic activation following a fully predicted reward at the time of outcome (i.e., the participant is fully aware to have pressed the button in time and expects his/her reward). Hypothetically, this dopamine activation helps to explain functional activation elicited by conditioned reward-indicating cues in the MID task. Figure modified and combined according to Knutson et al. (2001b) and Schultz (1998)

A modified version of the MID task as described by Knutson et al. (2001a, b) was used to study the BOLD response during anticipation and feedback of monetary reward (gain) and punishment (loss). The subjects’ monetary gain depended on their performance in a simple reaction time task where they had to respond while a target stimulus was presented (Fig. 1).

Each trial started with presentation of a cue indicating whether subjects could win money (circle), avoid losing money (square) or neither (neutral cue, triangle). The magnitude of the incentive (0.10 €; 0.60 €, or 3 €) was denoted by the number of horizontal lines (1, 2, or 3) inside the cue. Before scanning, participants were taught about the meaning of the seven different cues and that they would receive any earned money after the scanning session was completed. A training session without monetary reimbursement took place during the acquisition of the anatomical scan. Between cues and target, a variable delay (mean 3,990 ms) was inserted. After responding, feedback was given for 1,650 ms. Due to application of an adaptive algorithm for target duration, subjects succeeded on about 67 % of the trials. Hits (=success) were defined as button presses within the time frame of the target presentation (maximum 1 s). Subjects performed two sessions consisting of 54 gains, 54 losses, and 36 neutral trials, which were presented in a random sequence. Each run lasted about 14 min with a mean trial duration of approximately 7.69 s and a mean intertrial interval of 3.53 s.

### MRI scanning

Event-related fMRI was performed on a 1.5-T scanner (Magnetom VISION Siemens®) using gradient-echo echo-planar imaging (GE-EPI, repetition time (TR) = 1.9 s for the MID task, echo time (TE) = 40 ms, flip angle = 90°, matrix = 64 × 64) with a voxel size of 4 × 4 × 3.3 mm^3^. Eighteen slices were collected approximately parallel to the bicommissural plane (ac-pc plane). For anatomical reference, a 3D magnetization-prepared rapid gradient echo (MPRAGE; TR = 9.7 ms; TE = 4 ms; flip angle 12°, matrix = 256 × 256, voxel size 1 × 1 × 1 mm^3^) image data set was acquired.

### Data processing and analysis

Functional MRI data were analyzed with the Statistical Parametric Mapping 8 (SPM8) software package (http//www.fil.ion.ucl.ac.uk/spm) and the ArtRepair software developed by Mazaika and colleagues (2005) (http://cibsr.stanford.edu/tools/human-brain-project/artrepair-software.html). The ventral striatal, insula, and amygdala ROIs were specified from the publication-based probabilistic Montreal Neurological Institute (MNI) atlas used as binary masks at the threshold of 0.75 probability (please refer to http://hendrix.imm.dtu.dk/services/jerne/ninf/voi/index-alphabetic.html).

First, we used ArtRepair to correct images for technical artifacts. After discarding the first three volumes, we then performed slice time correction, realignment, spatial normalization into the MNI standard space, and smoothing with an 8-mm full-width-at-half-maximum Gaussian kernel. Patients and controls did not differ in their maximum, mean, and cumulative head motion (repeated measures ANOVA with time as intrasubject factor and group as between subject factor: all main effects and interaction *p* > 0.1). The preprocessed fMRI data was analyzed using the general linear model approach in a two-stage model. Due to significant group differences in age, gender, and smoking behavior (smokers/nonsmokers), those three variables were modeled as covariates throughout all analyses.

On the individual first level, the seven cue conditions, the target, and the five feedback conditions (successful gain, nonsuccessful gain, successful loss avoidance, nonsuccessful loss avoidance, neutral condition) were modeled separately as regressors after being convolved with a canonical hemodynamic response function (HRF). Realignment parameters were included as additional regressors. For the anticipation phase, the contrast images “gain *minus* neutral cues” and “loss avoidance *minus* neutral cues” were computed combining the three different values for gain and loss avoidance, respectively. On the second-level, between-group differences were assessed with separate full-factorial ANOVA designs for the two abovementioned individual contrast images using an F-contrast.

Given our strong a priori hypothesis for ventral striatal activation during the anticipation phase of the MID task (Knutson et al. 2001a, 2008), we used small-volume correction implemented in SPM 8 (significance level *p* < 0.05, family-wise error (FWE)-corrected). We also performed whole-brain analyses with a threshold of *p* < 0.05, FWE-corrected. For post hoc *t* tests, we conducted five different analyses in SPSS, comparing each diagnostic group to healthy controls.

### Correlations between fMRI signal and symptoms of depression and anxiety

Self-ratings of depressed mood (BDI) had been applied in all diagnostic groups except for patients with bipolar disorder (acute manic episode). Self-ratings of anxiety (STAI) had been applied in all groups except for patients with schizophrenia. In order to probe a relationship between VS activation and depressed mood/anxiety, the following analyses were performed:

We extracted the individuals’ mean parameter estimates from the cluster that showed a significant activation in the between-group F-contrasts “anticipation of gain minus neutral cues” and “anticipation of loss *minus* neutral cues.” First, in exploratory analyses, the mean parameter estimates were correlated with the subjects’ individual BDI and STAI scores across diagnostic categories. These exploratory correlation analyses were substantiated with partial correlation analyses controlling for diagnostic category, age, gender, and smoking behavior.

In a second step, we performed stepwise regression analyses to test whether ventral striatal activation predicted depressed mood or anxiety beyond the effects of diagnostic category, age, gender, and smoking behavior.

Finally, to ensure that effects were not driven by significant differences in the association between depressed mood/anxiety and ventral striatal activation between diagnostic categories, we computed an interaction term (specifically, by multiplying the categorical variable “diagnostic category” with the main predictor variable, ventral striatal activity) and added this interaction term to the regression model. Here, a significant effect for the interaction term would indicate a significant difference in the association between ventral striatal activity and depressed mood or anxiety between the diagnostic categories.

Throughout the analyses, gender was coded as “1” for males and “2” for females; smoking behavior was coded as “1” for “smoker” and “2” for “non-smoker”; and the different diagnostic categories were coded from “1” to “6” (1 = healthy controls, 2 = patients with alcohol dependence, 3 = patients with schizophrenia, 4 = patients with MDD, 5 = patients with bipolar disorder, acute manic episode, 6 = patients with ADHD). Due to the variety of substance classes, it was not possible to convert the individuals’ medication in a meaningful fashion; therefore, we added medication as a categorical covariate, with “0” for “no medication and “1” for “medication.”

## Results

### Behavioral data

Mean reaction times (RT) revealed a significant main effect of cue type (*F*_(2,174)_ = 101.23, *p* < 0.001), indicating faster responses during both gain and loss trials (RT gain>neutral, *t* = −11.89, *p* < 0.001; RT loss>neutral, *t* = −10.96, *p* < 0.001; and RT loss > RT gain, *t* = 2.11, *p* = 0.04). They also revealed a main effect of group (*F*_(5,174)_ = 4.81, *p* < 0.001), with patients having a diagnosis of schizophrenia showing significantly slower responses than patients with MDD, ADHD, and healthy controls (post hoc *t* tests all *p* < 0.007; for detailed results, please see supplement Table 2). Please note that the MID task is programmed to ensure equal percentages of gains and losses by adjusting to individual reaction times (Beck et al. 2009; Knutson et al. 2000). There was no group-by-cue interaction (*F*_(10,350)_ = 0.85, *p* > 0.5), and the reaction time patterns were very similar in all participants, indicating that all participants understood the paradigm and were engaged in the task.

### Brain activation

**Fig. 2:**
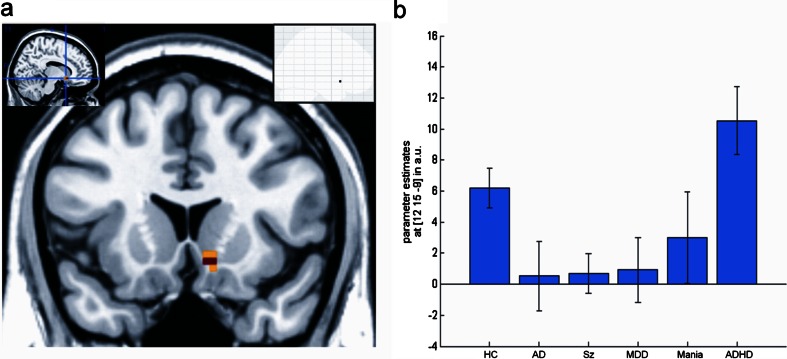
Difference in VS BOLD response during reward compared to neutral anticipation between diagnostic groups (see below). **a** Significant main effect of group in right VS ([*x y z*] = [12 15 − 9], *F* = 4.59, *p* = 0.008, FWE-corrected for VS-ROI; displayed at coronal section *y* = 15, red *p* < 0.001, yellow: *p* < 0.005; *left upper insert* sagittal section at *x* = 12; *right insert* glassbrain at *p* < 0.001, *k* > 2 showing the specificity of the finding). **b** Plot of the mean parameter estimates of this cluster. *HC*, healthy controls (*n* = 54); *AD*, alcohol-dependent patients (*n* = 26); *Sz*, schizophrenia patients (*n* = 44); *MDD*, major depressive disorder patients (*n* = 24); *Mania*, bipolar patients (acute manic episode; *n* = 13); *ADHD*, attention deficit/hyperactivity disorder patients (*n* = 23); *a.u.*, arbitrary units

When contrasting anticipation of gain versus a neutral condition, a significant effect of group was observed in the right VS ([*x y z*] = [12 15 − 9], *F* = 4.59, *p* = 0.008, FWE-corrected for VS-ROI), and trendwise in left VS ([*x y z*] = [−15 9 − 6], *F* = 3.36, *p* = 0.06, FWE-corrected for VS-ROI) (Fig. 2).

Post hoc *t* tests revealed a significant reduction in right VS activation during reward compared to neutral anticipation in patients with schizophrenia versus healthy controls (*t* = 2.68, *p* = 0.009). This effect was independent of schizophrenia patients’ antipsychotic medication (please see Table 1): a two-sample *t* test between medicated (*n* = 28; mainly with first generation antipsychotics) and unmedicated (*n* = 16; medication-free for at least four half-lives of the previously given medication) patients revealed no significant differences in right ventral striatal activation (*t* = −0.25, *p* = 0.80). We also found a significant reduction in right VS activation in patients with MDD compared to healthy controls (*t* = 2.32, *p* = 0.02) and in patients with alcohol dependence compared to healthy controls (*t* = 2.46, *p* = 0.03). There was no significant difference in right ventral striatal activation between healthy controls and patients with an acute manic episode (*t* = 0.95, *p* = 0.35) or between healthy controls and patients with ADHD (*t* = −1.46, *p* = 0.15). Whole-brain analyses revealed no further group differences outside the VS.

### Correlations between fMRI signal and symptoms of depression and anxiety

#### Anticipation of gain minus neutral cues

Participants with BDI did not differ significantly from participants with missing BDI scores with regard to gender (*F*_1_ = 0.15, *p* = 0.7), age (*F*_1_ = 0.001, *p* = 0.98), or smoking behavior (*F*_1_ = 0.15, *p* = 0.7). Patients with STAI did not differ significantly from patients without STAI scores regarding age (*F*_1_ = 3.7, *p* = 0.06) and smoking behavior (*F*_1_ = 0.61, *p* = 0.44); however, because STAI scores were not assessed in schizophrenia patients (who included a substantial number of women), more men than women had a STAI score (*F*_1_ = 7.25, *p* = 0.008).

In our exploratory analyses, we observed a significant negative bivariate correlation between right ventral striatal activation (at [*x y z*] = [12 15 − 9]) and depressive symptoms (Pearson’s *r* = −0.19, *p* = 0.03), which was also found in the partial correlation analyses controlling for age, gender, smoking behavior, and diagnostic category (correlation coefficient = −0.23, *p* = 0.008).

To ensure that this effect was not driven by differences in the direction or magnitude of the correlation between healthy controls and patients, we split our sample accordingly and repeated bivariate and partial correlation analyses. In bivariate correlations, we found correlation coefficients of similar magnitude and direction in both healthy controls (Pearson’s *r* = −0.16) and patients (Pearson’s *r* = −0.15), which, however, did not reach statistical significance (*p* = 0.14 and *p* = 0.09, respectively). We observed the same effect in partial correlations correcting for age, gender, smoking behavior, and diagnostic category in healthy controls (correlation coefficient = −0.15, *p* = 0.33) and patients (correlation coefficient = −0.16, *p* = 0.15). These results suggest that the lack of significance is due to decreased power and assert that no major difference in the magnitude or direction of the correlation between patients and controls generated the overall association.

**Fig. 3:**
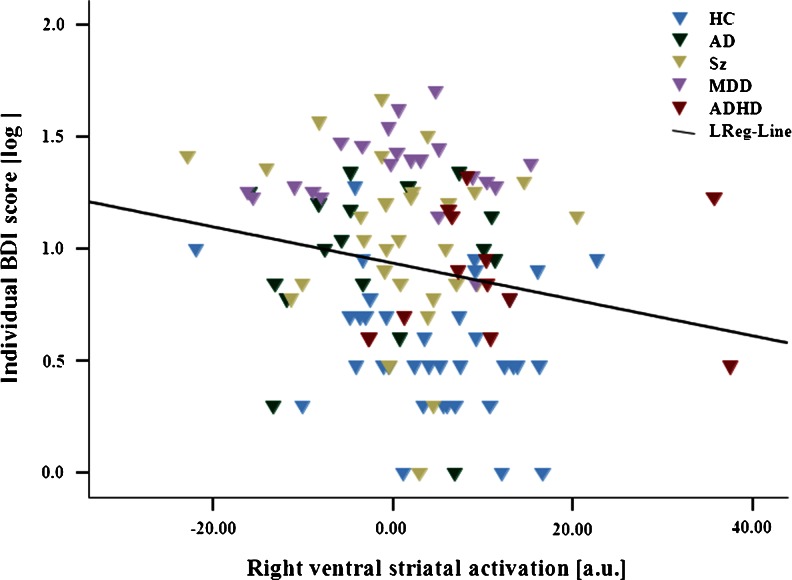
Correlation between fMRI signal and depressive symptoms during reward anticipation. Significant negative correlation between right VS reward anticipation signal at peak of group difference at [9 15 − 12] and depression symptom severity (log-transformed BDI; beta = −0.21, *p* = 0.01). *HC*, healthy controls (*n* = 49); *AD*, alcohol-dependent patients (*n* = 18); *Sz*, schizophrenia patients (*n* = 29); *MDD*, major depressive disorder patients (*n* = 21); *ADHD*, attention deficit/hyperactivity disorder patients (*n* = 15); *LReg-Line*, linear regression line across all subjects; *a.u.*, arbitrary units; *log*, log transformed.

In stepwise regression analysis, VS activations predicted depressed mood beyond the effects of diagnostic category, age, gender, and smoking behavior (beta = −0.20, *p* = 0.008). Since data on the BDI were skewed to the right given the inclusion of healthy subjects (floor effect), we log-transformed BDI sum score data. Again, results showed that VS reward anticipation signal explained variance above and beyond the effects of diagnostic category, age, gender, and smoking behavior (beta = −0.21, *p* = 0.01; Fig. 3). This effect also remained significant when we controlled for the effects of medication (beta = −0.21, *p* < 0.01). The application of the interaction term between diagnostic category and VS activity did not yield significant results (beta = −0.19, *p* = 0.31), indicating that different diagnostic groups did not differ significantly in the association between the reward anticipation signal in the right VS and depressive symptoms.

We found no significant correlation between right ventral striatal activation (at [*x y z*] = [12 15 − 9]) and anxiety, measured with the STAI (Pearson’s *r* = 0.03, *p* = 0.78). Therefore, no further analyses were applied.

#### “Anticipation of loss minus a neutral condition”

We did not observe a significant cluster of activation in the between-group F-contrast “anticipation of loss minus neutral cues.” Therefore, no further analyses were applied.

## Discussion

To the best of our knowledge, this is the first study assessing reward anticipation in adults across nosological boundaries in a range of different psychiatric disorders. First, we observed a significant reduction in right ventral striatal activation during reward anticipation in patients with schizophrenia compared to healthy controls. Ventral striatal hypoactivation was also recently reported by Nielsen et al. (2012) and Esslinger et al. (2012), who assessed reward anticipation in medication-free patients with schizophrenia or schizoaffective disorder. In line with this, reduced ventral striatal signaling was also observed during reversal learning in unmedicated schizophrenia patients (Schlagenhauf et al. 2014). Our own sample consists of unmedicated as well as medicated patients; however, post hoc *t* tests revealed no significant differences between the two subgroups. Our patients were mainly receiving first-generation antipsychotics such as haloperidol or flupentixol; this observation is in line with previous findings of our group (Schlagenhauf et al. 2008), where we found ventral striatal hypoactivation in schizophrenia patients receiving first-generation antipsychotics, which normalized after a change from first- to second-generation antipsychotics.

Secondly, we observed a reduction of right VS activation during reward anticipation in patients with major depression and in patients with alcohol dependence compared to healthy controls. The moderate reduction in ventral striatal activation in alcohol-dependent patients is in line with the more inconsistent literature on reward anticipation in alcohol dependence (Bjork et al. 2012; Wrase et al. 2007). Unmedicated MDD patients have also been reported to show nonsignificant or rather moderate reductions in VS activation during reward anticipation (Knutson et al. 2008; Pizzagalli et al. 2009; Smoski et al. 2009; Stoy et al. 2012). Diagnostic group differences in VS activation to reward-predicting cues can be due to different degrees and patterns of dopamine dysfunction. For example, in unmedicated schizophrenia, presynaptic dopamine release is elevated (Abi-Dargham et al. 2009), while in alcohol dependence, both dopamine release and D2 receptors appear to be reduced (Heinz et al. 2005b; Martinez et al. 2005). In major depression, serotonin medication normalized previously blunted VS activation during reward anticipation (Stoy et al. 2012), pointing to an interaction between serotonin and dopamine neurotransmitter systems in this affective disorder.

Patients with acute manic episode or ADHD did not show significant reductions in VS functional activation during reward anticipation compared with healthy controls. Previous studies reported mixed results regarding ventral striatal activation in ADHD, which was attributed to differences in psychopathological symptom severity or medication status (Carmona et al. 2012; Stoy et al. 2011).

No significant group differences were observed with respect to loss anticipation. This finding is in line with previous findings from our studies as well as others (Schlagenhauf et al. 2009; Stoy et al. 2011; Waltz et al. 2010). In cases where group differences in ventral striatal activation during loss anticipation between healthy controls and patients were found, they tended to be small (Beck et al. 2009; Wrase et al. 2007). This is in accordance with observations suggesting that in the MID task, functional activation elicited by reward anticipation (versus neutral cues) is stronger than activation elicited by loss anticipation (versus neutral cues) (Heinz and Schlagenhauf 2010; Knutson et al. 2001a). The MID task thus appears to be more suitable to assess ventral striatal correlates of gain anticipation.

Critically, and in accordance with a dimensional approach that cuts across nosological boundaries, we found a significant negative correlation between ventral striatal activation during reward anticipation and severity of depressive symptoms in all participants (with the sole exception of manic patients for whom no BDI scores were available). We did not find a correlation between anxiety symptom severity and neuronal activity during reward anticipation. These observations show that *across* nosological boundaries, depressive symptoms are a direct correlate of dysfunction of reward anticipation in several mental disorders, including schizophrenia. The BOLD response observed in our study reflects the impact of afferent inputs to the VS, which are in turn modulated by a multitude of neurotransmitters, most prominently dopamine (Knutson et al. 2004; Schlagenhauf et al. 2013; Schott et al. 2008). In accordance with this hypothesis, phasic increases in dopaminergic neurotransmission during reward anticipation have been associated with the attribution of motivational salience to rewards and reward-indicating cues as well as the positive mood that can accompany an expectation of reinforcement (Drevets et al. 2001; Hasler et al. 2008). A dysfunction of reward expectation can thus interfere with motivation and positive effect elicited by reward-predictive cues and, in our study, was directly correlated with the severity of depressive symptoms. Indeed, apathy and anhedonia, clinical correlations of (striatal) dopamine dysfunction (Gradin et al. 2011; Heinz et al. 1994), are key symptoms of depression (according to ICD-10 and DSM-IV). Anxiety, on the other hand, was not directly correlated with dysfunction of reward anticipation. Severity of anxiety has more often been associated with alterations in serotonergic modulation of the amygdala and other limbic areas (Hariri et al. 2002; Heinz et al. 2011). A dimensional approach can target such mood states and their hypothetically associated mechanisms (e.g., impaired anticipation and experience of reward and dopamine dysfunction) across traditional disorders. Further studies will have to explore the association between anxiety, learning from punishment, and serotonin dysfunction (Daw et al. 2002; Reimold et al. 2008; Robinson et al. 2012).

## Limitations

While all subjects were studied with the same scanner (1.5 T, Magnetom VISION Siemens®) and paradigms, patient groups—according to age differences in prevalence—were not matched for age and smoking behavior, variables which were modeled as covariates in all analyses. We did not observe significant gender differences; however, study conditions were not designed to assess gender or age differences, and further studies are required to explore such effects. Secondly, we measured dimensional correlates of reward anticipation in clinical disorders that we had previously assessed one by one, comparing them with healthy controls. Therefore, we had mainly acquired disorder-specific scores rather than applying a larger psychological test battery for all participants, which is a limitation of our study. In addition, other scales besides the BDI can be applied to measure depressive symptoms in more detail, as the BDI is a self-administered questionnaire with all potential biasing due to self-reports (Bowling 2005). Finally, the incorporation of further diagnostic groups known to involve the dopaminergic system (e.g., obsessive-compulsive disorder) would have been desirable; however, such data of further groups were not at our disposal.

Altogether, our observations show that reward anticipation is impaired in several psychiatric disorders, confirming the prominent role of reward prediction and learning for complex human behavior (Montague et al. 2004; O'Doherty et al. 2004; Schultz 2010; Tobler et al. 2007). Our study reveals differences between diagnostic groups, in line with a categorical approach toward psychiatric disorders. At the same time, our findings strengthen the idea that psychiatric disorders are complex phenotypes composed of patterns of symptoms (such as dysfunction of anticipation and experience of reward versus punishment) and suggest that symptoms with a comparable neurobiological signature (here, blunted VS activation) can be present in different psychiatric disorders and contribute to negative mood across nosological boundaries (Heinz 2002; Robbins et al. 2012; van Os and Kapur 2009). Therefore, our findings can help to explain why medication modulating, e.g., dopamine or serotonin neurotransmission, has benefits in a variety of disorders. The dimensional strategy, pursued, e.g., by the RDoC project (Cuthbert 2014; Insel et al. 2010), suggests that neurobiological research in psychiatric disorders can advance by targeting such core mechanisms that are implicated in diverse clinical entities. In the future, an integration of these approaches may help to focus on therapeutic strategies to clinically relevant syndromes within and across mental disorders.

## Electronic supplementary material

Below is the link to the electronic supplementary material.

ESM 1 (DOC 309 kb)
