# Supplementary material for: Dimensional psychiatry: reward dysfunction and depressive mood across psychiatric disorders
Source: Psychopharmacology (Berl). 2014 Jun 29;232(2):331–41. doi: 10.1007/s00213-014-3662-7 (PMC4297301; doi:10.1007/s00213-014-3662-7)
Supplement: Supplementary file 1 — (DOC 309 kb) [file 213_2014_3662_MOESM1_ESM.doc]

**SUPPLEMENT**

**METHODS**

**Data processing and analysis**

On the individual first-level, the seven cue conditions, the target and the five feedback conditions (successful gain, non-successful gain, successful loss-avoidance, non-successful loss-avoidance, neutral condition) were modeled separately as regressors after being convolved with a canonical hemodynamic response function (HRF). Realignment parameters were included as additional regressors.

The feedback phase was analyzed contrasting gain versus miss of gain (“successful gain *minus* non-successful gain”) and loss versus avoidance of loss (“non-successful loss-avoidance *minus* successful loss-avoidance”).

On the second-level, between-group differences were assessed with separate full-factorial ANOVA designs for the four above mentioned individual contrast images using an F-contrast. We performed whole brain analyses with a threshold of p<0.05, FWE–corrected.

**RESULTS**

**Brain activation**

**Correlations between fMRI signal and symptoms of depression and anxiety**

**“Anticipation of gain *minus* neutral cues”**

We also conducted BDI factor score analyses. These explorative analyses revealed a negative correlation between right ventral striatal activity during reward anticipation and the subitem “somatic factor” (; BDI-Items: 15, 16, 18, 19, 20; r=-0.23, p=0.009, n=132), and a weaker, albeit still significant negative correlation between right ventral striatal activity during reward anticipation and the subitem “cognitive-affective factor” (; BDI-Items: Items 1-14, 17, 21; r=-0.17, p=0.05, n=132).

**Feedback phase**

We did not observe a significant cluster of activation in the between-group F-contrast “non-successful loss-avoidance *minus* successful loss-avoidance” nor in the between-group F-contrast “non-successful loss-avoidance *minus* successful loss-avoidance”. Therefore, no further analyses were applied.

**REFERENCES**

Abler B, Greenhouse I, Ongur D, Walter H, Heckers S (2008) Abnormal reward system activation in mania. Neuropsychopharmacology 33: 2217-27

Abler B, Walter H, Erk S (2005) Neural correlates of frustration. Neuroreport 16: 669-72

Andrews MM, Meda SA, Thomas AD, Potenza MN, Krystal JH, Worhunsky P, Stevens MC, O'Malley S, Book GA, Reynolds B, Pearlson GD (2011) Individuals family history positive for alcoholism show functional magnetic resonance imaging differences in reward sensitivity that are related to impulsivity factors. Biol Psychiatry 69: 675-83

Beck A, Schlagenhauf F, Wustenberg T, Hein J, Kienast T, Kahnt T, Schmack K, Hagele C, Knutson B, Heinz A, Wrase J (2009) Ventral striatal activation during reward anticipation correlates with impulsivity in alcoholics. Biol Psychiatry 66: 734-42

Bermpohl F, Kahnt T, Dalanay U, Hagele C, Sajonz B, Wegner T, Stoy M, Adli M, Kruger S, Wrase J, Strohle A, Bauer M, Heinz A (2010) Altered representation of expected value in the orbitofrontal cortex in mania. Hum Brain Mapp 31: 958-69

Bjork JM, Smith AR, Chen G, Hommer DW (2012) Mesolimbic recruitment by nondrug rewards in detoxified alcoholics: effort anticipation, reward anticipation, and reward delivery. Hum Brain Mapp 33: 2174-88

Carmona S, Hoekzema E, Ramos-Quiroga JA, Richarte V, Canals C, Bosch R, Rovira M, Soliva JC, Bulbena A, Tobena A, Casas M, Vilarroya O (2012) Response inhibition and reward anticipation in medication-naive adults with attention-deficit/hyperactivity disorder: a within-subject case-control neuroimaging study. Hum Brain Mapp 33: 2350-61

Dichter GS, Kozink RV, McClernon FJ, Smoski MJ (2012) Remitted major depression is characterized by reward network hyperactivation during reward anticipation and hypoactivation during reward outcomes. J Affect Disord 136: 1126-34

Edel MA, Enzi B, Witthaus H, Tegenthoff M, Peters S, Juckel G, Lissek S (2013) Differential reward processing in subtypes of adult attention deficit hyperactivity disorder. J Psychiatr Res 47: 350-6

Hoogman M, Aarts E, Zwiers M, Slaats-Willemse D, Naber M, Onnink M, Cools R, Kan C, Buitelaar J, Franke B (2011) Nitric oxide synthase genotype modulation of impulsivity and ventral striatal activity in adult ADHD patients and healthy comparison subjects. Am J Psychiatry 168: 1099-106

Juckel G, Friedel E, Koslowski M, Witthaus H, Ozgurdal S, Gudlowski Y, Knutson B, Wrase J, Brune M, Heinz A, Schlagenhauf F (2012) Ventral striatal activation during reward processing in subjects with ultra-high risk for schizophrenia. Neuropsychobiology 66: 50-6

Juckel G, Schlagenhauf F, Koslowski M, Filonov D, Wustenberg T, Villringer A, Knutson B, Kienast T, Gallinat J, Wrase J, Heinz A (2006a) Dysfunction of ventral striatal reward prediction in schizophrenic patients treated with typical, not atypical, neuroleptics. Psychopharmacology 187: 222-8

Juckel G, Schlagenhauf F, Koslowski M, Wustenberg T, Villringer A, Knutson B, Wrase J, Heinz A (2006b) Dysfunction of ventral striatal reward prediction in schizophrenia. NeuroImage 29: 409-16

Knutson B, Bhanji JP, Cooney RE, Atlas LY, Gotlib IH (2008) Neural responses to monetary incentives in major depression. Biol Psychiatry 63: 686-92

Nielsen MO, Rostrup E, Wulff S, Bak N, Broberg BV, Lublin H, Kapur S, Glenthoj B (2012a) Improvement of Brain Reward Abnormalities by Antipsychotic Monotherapy in Schizophrenia. Arch Gen Psychiatry: 1-10

Nielsen MO, Rostrup E, Wulff S, Bak N, Lublin H, Kapur S, Glenthoj B (2012b) Alterations of the Brain Reward System in Antipsychotic Naive Schizophrenia Patients. Biological psychiatry.

Pizzagalli DA, Holmes AJ, Dillon DG, Goetz EL, Birk JL, Bogdan R, Dougherty DD, Iosifescu DV, Rauch SL, Fava M (2009) Reduced caudate and nucleus accumbens response to rewards in unmedicated individuals with major depressive disorder. Am J Psychiatry 166: 702-10

Schiller CE, Minkel J, Smoski MJ, Dichter GS (2013) Remitted major depression is characterized by reduced prefrontal cortex reactivity to reward loss. J Affect Disord 151: 756-62

Schlagenhauf F, Juckel G, Koslowski M, Kahnt T, Knutson B, Dembler T, Kienast T, Gallinat J, Wrase J, Heinz A (2008) Reward system activation in schizophrenic patients switched from typical neuroleptics to olanzapine. Psychopharmacology 196: 673-84

Schlagenhauf F, Sterzer P, Schmack K, Ballmaier M, Rapp M, Wrase J, Juckel G, Gallinat J, Heinz A (2009) Reward feedback alterations in unmedicated schizophrenia patients: relevance for delusions. Biol Psychiatry 65: 1032-9

Simon JJ, Biller A, Walther S, Roesch-Ely D, Stippich C, Weisbrod M, Kaiser S (2010) Neural correlates of reward processing in schizophrenia--relationship to apathy and depression. Schizophr Res 118: 154-61

Smoski MJ, Rittenberg A, Dichter GS (2011) Major depressive disorder is characterized by greater reward network activation to monetary than pleasant image rewards. Psychiatr Res 194: 263-70

Stoy M, Schlagenhauf F, Schlochtermeier L, Wrase J, Knutson B, Lehmkuhl U, Huss M, Heinz A, Strohle A (2011) Reward processing in male adults with childhood ADHD--a comparison between drug-naive and methylphenidate-treated subjects. Psychopharmacology 215: 467-81

Stoy M, Schlagenhauf F, Sterzer P, Bermpohl F, Hagele C, Suchotzki K, Schmack K, Wrase J, Ricken R, Knutson B, Adli M, Bauer M, Heinz A, Strohle A (2012) Hyporeactivity of ventral striatum towards incentive stimuli in unmedicated depressed patients normalizes after treatment with escitalopram. J Psychopharmacol 26: 677-88

Strohle A, Stoy M, Wrase J, Schwarzer S, Schlagenhauf F, Huss M, Hein J, Nedderhut A, Neumann B, Gregor A, Juckel G, Knutson B, Lehmkuhl U, Bauer M, Heinz A (2008) Reward anticipation and outcomes in adult males with attention-deficit/hyperactivity disorder. NeuroImage 39: 966-72

Waltz JA, Schweitzer JB, Ross TJ, Kurup PK, Salmeron BJ, Rose EJ, Gold JM, Stein EA (2010) Abnormal responses to monetary outcomes in cortex, but not in the basal ganglia, in schizophrenia. Neuropsychopharmacology 35: 2427-39

Whisman MA, Perez JE, Ramel W (2000) Factor structure of the Beck Depression Inventory-Second Edition (BDI-II) in a student sample. J Clin Psychol 56: 545-51

Wrase J, Schlagenhauf F, Kienast T, Wustenberg T, Bermpohl F, Kahnt T, Beck A, Strohle A, Juckel G, Knutson B, Heinz A (2007) Dysfunction of reward processing correlates with alcohol craving in detoxified alcoholics. NeuroImage 35: 787-94

**Supplementary Table 1**

| **Disorder** | **Authors** | | **Title** | **Participants** | **Medication status** | **Monetary Incentive Delay Task (MID-Task)** | **Ventral striatal (VS)/Nucleus accumbens (NAcc) activation during reward anticipation** |
| --- | --- | --- | --- | --- | --- | --- | --- |
| **Bipolar Affective Disorder;**  **Acute manic/mixed/**  **hypomanic episode;**  **Schizophrenia;**  **Schizoaffective Disorder** | | **Abler B.**  Neuropsycho  pharmacology | **Abnormal Reward System Activation in Mania** | **12 patients** with acute manic episode of bipolar disorder *(7 males)*.  **12 patients** with current episode of schizoaffective disorder or schizophrenia *(5 males).*  **12 HC** *(7 males)*. | All patients: **antipsychotic medication (1^st^ or 2^nd^ generation);**  also **mood stabilizers** (all bipolar, 8 schizophrenia patients), **antidepressants** (7 schizophrenia patients), **benzodiazepines**  (3 schizophrenia, 5 bipolar patients).  HC: **no medication.** | MID-Task by Abler . | Anticipation of *high gain or high loss > neutral*:  **No significant group differences.**  (Schizophrenia/Schizoaffective Patients vs HC/  Bipolar Patients vs HC/  Schizophrenia/Schizoaffective Patients vs Bipolar Patients) |
| **Schizophrenia** | | **Juckel G.**  NeuroImage | **Dysfunction of ventral striatal reward prediction in schizophrenia** | **10 patients** with schizophrenia.  **10 HC**.  *All males.* | All: **no medication.** | MID-task according to Knutson.  *(See “Methods” of the Manuscript).* | Anticipation of *gain or loss >neutral*,  Patients vs HC:  **Left VS ↓ in patients.** |
| **Schizophrenia** | | **Juckel G.**  Psycho  pharmacology | **Dysfunction of ventral striatal reward prediction in schizophrenic patients treated with typical, not atypical, neuroleptics** | **20 patients** with schizophrenia *(14 males*).  **10 HC** *(8 males)*. | 10 patients:  **1^st^ generation antipsychotics** *(8 males)*.  10 patients:  **2^nd^ generation antipsychotics** *(6 males).*  HC: **no medication.** | MID-task according to Knutson.  *(See “Methods” of the Manuscript).* | Anticipation of *gain > neutral*,  Patients with 1^st^ OR 2^nd^ generation antipsychotics vs HC:  **Left VS ↓ in patients with 1^st^ generation antipsychotics.**  **No group differences between HC and patients with 2^nd^ generation antipsychotics.** |
| **Ultra-High Risk**  **(UHR) for**  **Schizophrenia** | | **Juckel G.**  Neuro  psychobiology | **Ventral Striatal Activation during**  **Reward Processing in Subjects with**  **Ultra-High Risk for Schizophrenia** | **13 patients** with prodromal schizophrenia.  **13 HC.**  *11 males/group.* | 7 patients:  **Drug-naïve.**  6 patients: few days of **2^nd^ generation antipsychotic** treatment.  HC: **no medication.** | MID-task according to Knutson.  *(See “Methods” of the Manuscript).* | Anticipation of *gain > neutral*,  UHR-Patients vs HC:  **No significant group difference.**  Anticipation of *loss > neutral*:  Patients vs HC:  **Left VS ↓ in patients (Trend).** |
| **Schizophrenia;**  **Schizoaffective Disorder** | | **Nielsen M.**  Biological Psychiatry | **Alterations of the Brain Reward System in Antipsychotic Naïve Schizophrenia Patients** | **31** **schizophrenia patients** (including schizoaffective disorder), antipsychotic-naïve.  **31 HC.**  *22 males/group.* | Patients: **no medication 12h before scanning**. 7 benzodiazepines or sleeping medication, ***never* antipsychotic medication or Ritalin.**  HC: **no medication.** | Modified “Knutson-Task”. | Anticipation of *salient cues**,  Patients vs HC:  **Bilateral VS ↓ in patients.** |
| **Schizophrenia** | | **Nielsen M.**  JAMA Psychiatry | **Improvement of Brain Reward Abnormalities**  **by Antipsychotic Monotherapy in Schizophrenia** | **23 schizophrenia patients** (including schizoaffective disorder), antipsychotic-naïve *(16 males*).  **24 HC** *(20 males*). | Patients:  **T1:** **no medication 12h before scanning**. 7 benzodiazepines or sleeping medication, ***never* antipsychotic medication or Ritalin.**  **T2: Amisulpride,** treatment for 6 weeks, stable does for at least 2 weeks.  HC: **no medication.** | Modified “Knutson-Task”. | **T1 (antipsychotic naïve patients):**  Anticipation of *salient cues**,  Patients vs HC:  **Bilateral VS ↓ in patients.**  **T2 (Amisulpride for 6 weeks):**  Anticipation of *salient cues**,  Patients vs HC:  **No significant group difference.** |
| **Schizophrenia** | | **Schlagen-hauf F.**  Biological Psychiatry | **Reward Feedback Alterations in Unmedicated**  **Schizophrenia Patients: Relevance for Delusions** | **15** **patients** with schizophrenia.  **15 HC**.  *12 males/group.* | All: **no medication.** | MID-task according to Knutson.  *(See “Methods” of the Manuscript).* | Anticipation of *gain > neutral*,  Patients vs HC:  **Right VS ↓ in patients.**  **Left VS ↓ in patients (Trend).**  Anticipation of *loss > neutral*:  Patients vs HC:  **No significant group difference.** |
| **Schizophrenia** | | **Schlagen-hauf F.**  Psycho  pharmacology | **Reward system activation in schizophrenic patients switched from typical neuroleptics to olanzapine** | **10 patients** with schizophrenia.  **10 HC**.  *9 males/group*. | Patients at time point 1 (T1):  **1^st^ generation antipsychotics.**  Patients at time point 2 (T2):  Switch to **Olanzapine.**  HC: **No Medication.** | MID-task according to Knutson.  *(See “Methods” of the Manuscript).* | **T1 (1^st^ generation antipsychotics):**  Anticipation of *gain or loss >neutral*,  Patients vs HC:  **Right VS ↓ in patients.**  **T2 (Olanzapine):**  Anticipation of *gain or loss>neutral*,  Patients vs HC:  **No significant group difference.** |
| **Schizophrenia** | | **Simon J.J.**  Schizophrenia Research | **Neural correlates of reward processing in schizophrenia — Relationship to apathy and depression** | **15 patients** with schizophrenia.  **15 HC**.  *10 males/group.* | All patients: **2^nd^ generation antipsychotics**, 5 additionally with **antidepressants** or **mood stabilizers.**  HC: **no medication.** | MID-task according to Abler, Knutson. | Anticipation of *1€ or 0.2€>neutral,*  Patients vs HC:  **No significant group difference.** |
| **Schizophrenia** | | **Waltz J.A.**  Neuropsycho  pharmacology | **Abnormal Responses to Monetary Outcomes in Cortex, but not in the Basal Ganglia, in Schizophrenia** | **17 patients** with schizophrenia *(13 males)*.  **17 HC** *(12 males).* | All patients: **Stable antipsychotic**  **medication** (no changes for 4 weeks), almost  all with 2^nd^ generation antipsychotics.  HC: **no medication.** | Modified “Knutson-Task”. | Anticipation of *gain or loss >neutral,*  Patients vs HC:  **No significant group differences.**  *(Patients and HC: main effects of cue valence and cue magnitude on activity in bilateral VS.)* |
| **Alcohol**  **dependence** | | **Beck A.**  Biological Psychiatry | **Ventral Striatal Activation During Reward Anticipation**  **Correlates with Impulsivity in Alcoholics.** | **19 detoxified patients** with alcohol dependence.  **19 healthy controls (HC).**  *All males.* | All: **no medication.** | MID-task according to Knutson.  *(See “Methods” of the Manuscript).* | Anticipation of *gain or loss >neutral,*  Patients vs HC:  **Right VS ↓ in patients.** |
| **Alcohol**  **dependence** | | **Bjork J.M.**  Human Brain Mapping | **Mesolimbic Recruitment by Nondrug Rewards in**  **Detoxified Alcoholics: Effort Anticipation, Reward**  **Anticipation, and Reward Delivery** | **29 detoxified patients** *(15 males)* with alcohol dependence.  **23 HC** *(12 males)*. | All: **no medication.** | - Gain-indicating cues (0$, 1$, 10$)  - After target: notification whether success would be indicated by delivery of monetary reward (‘‘Win?’’)  or by lexical notification  - Outcome: Money or “+/- 0$” plus total amount. | Anticipation of *gain > neutral,*  Patients vs HC:  **No significant group differences.**  Anticipation of *“WIN”gain > “HIT”gain,*  Patients vs HC:  **No significant group differences.** |
| **Alcohol**  **dependence** | | **Wrase J.**  NeuroImage | **Dysfunction of reward processing correlates with alcohol**  **craving in detoxified alcoholics.** | **16 detoxified patients** with alcohol dependence.  **16 HC**.  *All males*. | All: **no medication.**  (Patients: no benzodiazepines or clomethiazol for 1 week) | MID-task according to Knutson.  *(See “Methods” of the Manuscript).* | Anticipation of *gain or loss >neutral*,  Patients vs HC:  **Left VS ↓ in patients.** |
| **Relatives of**  **patients with alcohol**  **dependence** | | **Andrews M.M.**  Biological Psychiatry | **Individuals Family History Positive for Alcoholism Show Functional Magnetic Resonance Imaging Differences in Reward Sensitivity That Are Related to Impulsivity Factors** | **19 HC with positive family history for alcoholism** (father alcohol dependent plus one or more one- or second-degree relatives) *(6 males)*.  **30 HC with negative family history for alcoholism** *(11 males)*. | ***n.a.***  probably: **no medication.** | “Modified Knutson-Task” → - Original symbols replaced by words (e.g. “Win $ 5”) | Anticipation of *5$,*  HC with positive family history vs HC with negative family history:  **Bilateral VS (NAcc) ↓in HC with positive family history.** |
| **Major Depressive Disorder** | | **Knutson B.**  Biological Psychiatry | **Neural Responses to Monetary Incentives in Major Depression** | **14 patients** with MDD *(5 males)*.  **12 HC** *(4 males)*. | All: **no medication.** | MID-Task  (Knutson 2001).  → “Knutson-Task” | Anticipation of *gain > neutral,*  Patients vs HC:  **No significant group differences.**  *(Patients: increasing activation of anterior cingulate cortex (ACC) with increasing amount of gain.*  *HC: increasing activation of ACC with increasing amount of loss.)* |
| **Major Depressive Disorder** | | **Pizzagalli D.A.**  American Journal of Psychiatry | **Reduced Caudate and Nucleus Accumbens Response to Rewards in Unmedicated Individuals With Major Depressive Disorder** | **30** **patients** with MDD *(17 males)***.**  **31 HC** *(16 males).* | All: **no medication.**  Patients: no psychotropic medication in the past 2 weeks, no fluoxetine in the past 6 weeks, no dopaminergic drugs or neuroleptics in the past 6 months. | Modified “Knutson Task”. | Anticipation of *gain > neutral,*  Patients vs HC:  **No significant group differences.**  (*Left posterior putamen relatively* ***↓*** *in patients.)* |
| **Major Depressive Disorder** | | **Smoski M.J.**  Psychiatry Research | **Major depressive disorder is characterized by greater reward network activation to monetary than pleasant image rewards** | **9 patients** with MDD.  **13 HC.** | **Patients:** 4 taking SSRI or SNRI  **HC:** no psychotropic medication | Modified “Knutson Task”. | Anticipation of *gain* or of  *pleasant pictures > neutral,*  Patients vs HC:  **No significant group differences in VS.** |
| **Major Depressive Disorder** | | **Stoy M.**  Journal of Psychopharmacology | **Hyporeactivity of ventral striatum towards incentive stimuli in unmedicated depressed patients normalizes after treatment with escitalopram.** | **15 patients** with MDD.  **15 HC**.  *10 males/group.* | Patients at T1:  **No medication.**  Patients at T2:  After 6 weeks of open-label treatment with **Escitalopram.**  HC: **No Medication.** | MID-task according to Knutson.  *(See “Methods” of the Manuscript).* | **T1 (no medication):**  Anticipation of *gain or loss>neutral*,  Patients vs HC:  **Right VS ↓ in patients *(anticipation of gain).***  **Bilateral VS ↓ in patients *(anticipation of loss).***  **T2 (Escitalopram):**  Anticipation of *gain or loss>neutral*,  Patients vs HC:  **No significant group difference.** |
| **Major Depressive Disorder,**  **remitted** | | **Dichter G.S.**  Journal of Affective Disorders | **Remitted major depression is characterized by reward network**  **hyperactivation during reward anticipation and hypoactivation during**  **reward outcomes** | **19 remitted patients** with MDD (rMDD) *(4 males).*  **19 HC** *(7 males).* | All: **no medication.**  Patients: 5 had received psychotropic medication in the past. | Modified “Knutson-Task”. | Anticipation of *gain > no-gain,*  Patients vs HC:  **No significant group differences in VS.** |
| **Major Depressive Disorder,**  **remitted** | | **Schiller C.E.**  Journal of Affective Disorders | **Remitted major depression is characterized by reduced prefrontal cortex reactivity to reward loss** | **19 remitted patients** with MDD (rMDD) *(4 males).*  **19 HC** *(7 males).* | All: **no medication in the past months.** | Modified “Knutson-Task”. | Anticipation of *loss > no-loss,*  Patients vs HC:  **No significant group differences in VS.** |
| **Bipolar affective disorder -**  **Acute manic**  **episode** | | **Bermpohl F.**  Human Brain Mapping | **Altered Representation of Expected Value in the Orbitofrontal Cortex in Mania.** | **15 patients** with acute mania *(8 males).*  **26 HC** *(15 males)*. | All patients received medication.  **(Mood stabilizers and/or benzodiazepines and/or antipsychotics).**  HC: **no medication.** | MID-task according to Knutson.  *(See “Methods” of the Manuscript).* | Anticipation of *gain > neutral,*  Patients vs HC:  **No significant group differences.**  Anticipation of *“expected value” (magnitude x valence),*  Patients vs HC:  **No significant group differences in VS.**  *(Left OFC* ***↑*** *in patients during anticipation of “expected value”.)* |
| **ADHD** | | **Carmona S.**  Human Brain Mapping | **Response Inhibition and Reward Anticipation in**  **Medication-Naϊve Adults With**  **Attention-Deficit/Hyperactivity Disorder: A**  **Within-Subject Case-Control Neuroimaging Study** | **19 patients** with ADHD*.*  **19 HC**.  *All males.* | All: **no medication.** | Modified “Knutson-Task”. | Anticipation of *gain > neutral,*  Patients vs HC:  **Bilateral VS ↓ in patients.** |
| **ADHD** | | **Edel M.-A.**  Journal of Psychiatric Research | **Differential reward processing in subtypes of adult attention deficit hyperactivity**  **disorder** | **24 patients with ADHD**  (**12** ADHD-**combined** type, **12** ADHD- **inattentive** type)**.**  **12 HC.**  *All males.* | All: **drug-naive.** | MID-Task according to Knutson. | Anticipation of *gain or loss >neutral*,  ADHD-inattentive type vs HC/  ADHD-inattentive type vs ADHD-combined:  **Bilateral VS ↓ in ADHD-inattentive type.**  ADHD-combined type vs HC:  **No significant group difference in VS.** |
| **ADHD** | | **Hoogman M.**  American Journal of Psychiatry | **Nitric Oxide Synthase Genotype Modulation of**  **Impulsivity and Ventral Striatal Activity in Adult ADHD Patients and Healthy Comparison Subjects.** | **63 patients** with adult ADHD *(26 males)*.  **41 HC** *(18 males).* | 19 patients: **Medication-naïve.**  44 patients: **Methylphenidate**  or **dextroamphetamine**  or **atomoxetine.**  All: **No medication 24h prior to testing.**  HC: **No medication.** | Modified “Knutson-Task”. | Anticipation of *gain > neutral,*  Patients vs HC:  **Bilateral VS ↓ in patients.** |
| **ADHD** | | **Stoy M.**  Psychopharmacology | **Reward processing in male adults with childhood**  **ADHD—a comparison between drug-naïve**  **And methylphenidate-treated subjects.** | **12 patients** with ADHD (**drug-naïve**, 7 remitted, 5 with adult ADHD).  **11 patients** with ADHD (**treatment** with methylphenidate (MPH) **during childhood** (6 remitted, 5 with adult ADHD).  **12 HC**.  *All males*. | All: **no medication.** | MID-task according to Knutson.  *(See “Methods” of the Manuscript).* | Anticipation of *gain or loss >neutral*,  Drug-naïve Patients vs HC/  Childhood-treatment Patients vs HC/  Drug-naïve Patients vs childhood-treatment Patients:  **No significant group difference in VS.** |
| **ADHD** | | **Stroehle A.**  NeuroImage | **Reward anticipation and outcomes in adult males with**  **attention-deficit/hyperactivity disorder.** | **10 patients** with adult ADHD.  **10 HC**.  *All males*. | All: **no medication.** | MID-task according to Knutson.  *(See “Methods” of the Manuscript).* | Anticipation of *gain > neutral,*  Patients vs HC:  **Right VS ↓ in patients.** |
| *HC = Healthy Controls, ADHD = Attention deficit/hyperactivity disorder.*  ** “Overall salience” = arousing events split into “behavioral salience” (predicted reward requires performance) and “valence anticipation” (anticipation of a monetarily significant outcome). Here: reduced activation most pronounced for behavioral salience, not significant for value anticipation.* | | | | | | | |

**Supplementary Table 2 – Reaction times across diagnostic groups and cue values**

|  | **Healthy controls** | | **Alcohol dependence** | | **Schizophrenia** | | **Major depressive**  **disorder** | | **Bipolar disorder**  **Acute manic episode** | | **Attention**  **deficit/hyperactivity disorder** | |
| --- | --- | --- | --- | --- | --- | --- | --- | --- | --- | --- | --- | --- |
| **Cue** | **Mean RT** | **SEM** | **Mean RT** | **SEM** | **Mean RT** | **SEM** | **Mean RT** | **SEM** | **Mean RT** | **SEM** | **Mean RT** | **SEM** |
| **-3 €** | 274 | 18 | 284 | 25 | 375 | 19 | 239 | 26 | 300 | 35 | 263 | 26 |
| **-0.6€** | 279 | 17 | 289 | 23 | 372 | 18 | 244 | 24 | 306 | 33 | 280 | 25 |
| **-0.1€** | 283 | 17 | 296 | 24 | 378 | 18 | 243 | 25 | 309 | 34 | 276 | 25 |
| **0 €** | 329 | 17 | 324 | 24 | 403 | 18 | 287 | 25 | 355 | 34 | 308 | 26 |
| **+0.1 €** | 281 | 16 | 288 | 23 | 370 | 17 | 246 | 24 | 307 | 32 | 270 | 24 |
| **+0.6 €** | 280 | 17 | 296 | 24 | 374 | 18 | 240 | 25 | 303 | 34 | 266 | 25 |
| **+3 €** | 269 | 17 | 283 | 24 | 367 | 18 | 235 | 25 | 292 | 34 | 256 | 25 |

*RT = Reaction time across all subjects of the specific group; SEM = Standard Error Mean*

**SUPPLEMENTARY FIGURE 1**

**Supplementary Fig. 1: Mean reaction time across diagnostic groups for different cue values**

*HC = Healthy controls (N=54); AD = Alcohol dependent patients (N=26); Sz = Schizophrenia patients (N=44); MDD = Major depressive disorder patients (N=24); Mania = bipolar patients (acute manic episode; N=13); ADHD = Attention deficit/hyperactivity disorder patients (N=23).*
